# Hxt13, Hxt15, Hxt16 and Hxt17 from *Saccharomyces cerevisiae* represent a novel type of polyol transporters

**DOI:** 10.1038/srep23502

**Published:** 2016-03-21

**Authors:** Paulina Jordan, Jun-Yong Choe, Eckhard Boles, Mislav Oreb

**Affiliations:** 1 Institute of Molecular Biosciences, Goethe University, Max-von-Laue Straße 9, 60438 Frankfurt am Main, Germany; 2 Department of Biochemistry and Molecular Biology, Rosalind Franklin University of Medicine and Science, The Chicago Medical School, 3333 Green Bay Road, North Chicago, IL, 60064, USA

## Abstract

The genome of *S. cerevisae* encodes at least twenty hexose transporter-like proteins. Despite extensive research, the functions of Hxt8-Hxt17 have remained poorly defined. Here, we show that Hxt13, Hxt15, Hxt16 and Hxt17 transport two major hexitols in nature, mannitol and sorbitol, with moderate affinities, by a facilitative mechanism. Moreover, Hxt11 and Hxt15 are capable of transporting xylitol, a five-carbon polyol derived from xylose, the most abundant pentose in lignocellulosic biomass. Hxt11, Hxt13, Hxt15, Hxt16 and Hxt17 are phylogenetically and functionally distinct from known polyol transporters. Based on docking of polyols to homology models of transporters, we propose the architecture of their active site. In addition, we determined the kinetic parameters of mannitol and sorbitol dehydrogenases encoded in the yeast genome, showing that they discriminate between mannitol and sorbitol to a much higher degree than the transporters.

Polyols, also called sugar alcohols, are present in all kingdoms of life. It is estimated that approximately one third of the global primary production by terrestrial plants and algae goes through polyol synthesis^1^. Polyols are produced by reduction of sugars in response to osmotic or drought stress, but are also used in several applications in food and pharmaceutical industries^2^. Mannitol is the most abundant polyol in nature, found in many bacteria, fungi, algae, lichens and plants^1^. For example, brown seaweed biomass, which is a promising substrate for microbial production of biofuels and chemicals^3^, consists of up to 40% mannitol. A significant amount of sorbitol is synthesized by some plants, especially *Rosaceae* species bearing stone fruits, such as cherries or plums, where sorbitol is the major carbohydrate produced^1^,^4^. This ubiquitous occurrence may explain why genes for utilization of polyols are found across many microbial phyla, although they are normally not used as a preferred carbon and energy source. The assimilation of mannitol and sorbitol is initiated by their oxidation to fructose, which is subsequently phosphorylated and funneled into the normal glycolytic route. Since a surplus NADH is formed in the oxidation step, their metabolism is strictly dependent on aerobic conditions.

Most *S. cerevisiae* strains grow on mannitol and sorbitol only after long adaptation, if at all^5^,^6^. While mannitol and sorbitol dehydrogenase activities could be measured in adapted strains^5^,^6^, the identity of transporters responsible for uptake of hexitols is unknown. Yeast genome encodes twenty members of the hexose transporter (Hxt) family, including the galactose permease Gal2 and glucose sensors Rgt2 and Snf3. Hxt1-Hxt7 are well described as hexose transporters (for review see refs 7, 8, 9), but the true physiological function of Hxt8-Hxt17 is unclear, since most of them show only a minor hexose transport activity^10^. In a recent study, aiming to engineer *S. cerevisae* for fermentation of algal biomass, *HXT13* and *HXT17* genes, encoding hexose transporter-like proteins, as well as annotated mannitol dehydrogenase (MDH) genes *DSF1* and YNR073C were found to be upregulated when yeast was adapted to grow on mannitol^11^. Interestingly, *HXT13* is co-localized with *DSF1* and *HXT17* with *YNR073C* in subtelomeric regions of chromosomes V and XIV, respectively. The authors showed that overexpression of *HXT13* or *HXT17* together with one of the MDH genes is necessary to confer growth of a yeast strain on mannitol. In a further recent work, it was found that dysfunction of the Tup1-Cyc8 corepressor complex is sufficient to relieve the repression of genes required for mannitol assimilation in *S. cerevisiae*^12^. Among the most prominently upregulated genes in *tup1-cyc8* mutants, the authors found *HXT10*, another hexose transporter-like protein of unknown function and speculated that it might act as a mannitol permease. However, this notion was not experimentally tested. In line with the preceding study^11^, *HXT13* and *HXT17* were also upregulated, but to a much lower extent compared to *HXT10* (and also less than mannitol dehydrogenase *DSF1*). Moreover, the transcription of other *HXT* genes with poorly defined function – *HXT8*, *HXT9*, *HXT11*, *HXT15*/*HXT16* - was also increased, suggesting a common regulatory mechanism. Due to the overlapping transcriptional pattern, the individual role of Hxt8-Hxt17 as potential polyol transporters was not clear. The assessment of their true function is additionally complicated by the fact that Hxt9, Hxt11 and Hxt13 were earlier implicated in drug resistance processes^13^,^14^. Therefore, we systematically investigated the ability of Hxt8-Hxt17 proteins to transport mannitol, sorbitol and xylitol in strains devoid of all known hexose transporter family members.

## Results

### Growth of *hxt* ^*0*^ strains overexpressing different transporters on polyols

**Figure 1:**
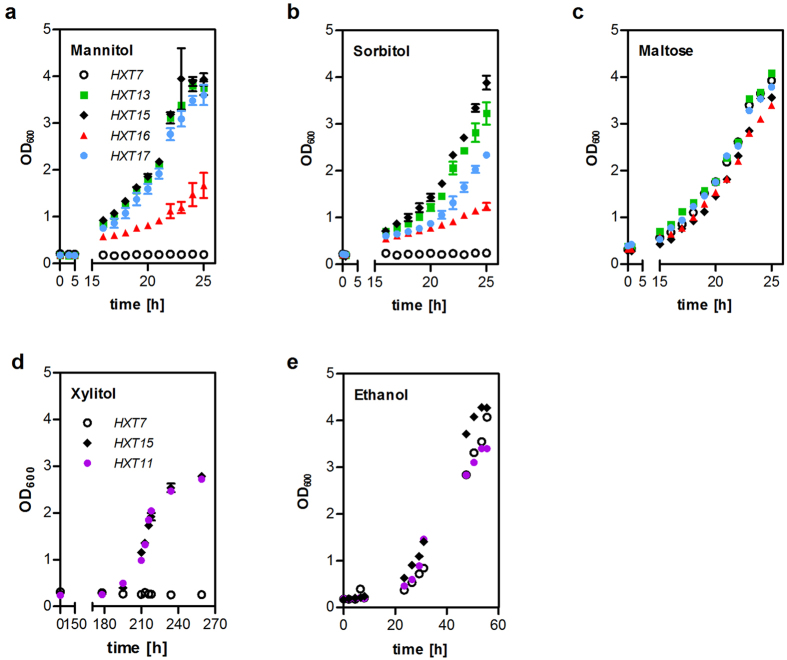
Growth of the *hxt0* strains expressing individual transporters on polyols. SC-media supplemented with 2% (w/v) of the indicated carbon source were inoculated in shake flasks with EBY.VW4000 (**a**–**c**) or AFY10 (**d**,**e**) cells expressing individual transporters together with the mannitol dehydrogenase YNR073C (**a**), sorbitol dehydrogenase *SOR1* (**b**,**c**) or xylitol dehydrogenase *XYL2* (**d**,**e**). Growth was monitored by measuring OD_600_ of the cultures. For growth on polyols (**a**,**b**,**d**), mean values and standard deviation of biological triplicates are shown. The controls (**c**,**e**) represent single measurements. Note that time-axes are truncated for clarity.

In order to assess the ability of individual Hxt proteins for polyol transport unambiguously, we performed a growth-based pre-screening in strains devoid of all known hexose transporters. For growth on mannitol and sorbitol, the strain EBY.VW4000^10^ was transformed with plasmids encoding Hxt1, Hxt4, Hxt7, Hxt8, Hxt9, Hxt10, Hxt11, Hxt13, Hxt14, Hxt15, Hxt16 or Hxt17 and appropriate polyol dehydrogenases (YNR073C for mannitol or Sor1 for sorbitol). Hxt1, Hxt4 and Hxt7 are representatives of low, medium and high affinity hexose transporters, respectively, and were used as controls. Of all transporters tested, only Hxt13, Hxt15, Hxt16 and Hxt17 conferred growth on mannitol and sorbitol (Supplementary Fig. S3), but only very weak growth on glucose (Supplementary Fig. S4). To determine the maximum growth rates (μ_max_), the strains expressing these four transporters or Hxt7 as a negative control were inoculated into liquid synthetic complete (SC) media supplemented with 2% (w/v) mannitol or sorbitol (Fig. 1a,b). The ability of the cells to grow was controlled on maltose-containing medium (Fig. 1c), as the utilization of this carbon source is not dependent on hexose transporters. Hxt13, Hxt15 and Hxt17 confer growth rates on polyols comparable to those on maltose (Fig. 1a–c). Only the strain expressing Hxt16 exhibited a significantly slower growth.

Growth tests on xylitol were performed in AFY10, an EBY.VW4000 derivative that was previously engineered for transporter screening on xylose^15^. Conversion of xylitol to xylulose was achieved by plasmid-based overexpression of the xylitol dehydrogenase *XYL2*^16^. To eliminate any limitations in the downstream pathway^17^,^18^, the strain overexpresses xylulokinase (*XKS1*), D-ribulose-5-phosphate epimerase (*RPE1*), D-ribulose-5-phosphate ketol-isomerase (*RKI1*), transketolase (*TKL1*) and transaldolase (*TAL1*) from a cassette integrated into the *PYK2* locus^15^. In this strain background, among all transporters tested, only Hxt11 and Hxt15 could support growth on agar plates containing xylitol (Supplementary Fig. S3). Again, the specific growth rates were determined on liquid media supplemented with 2% (w/v) xylitol whereby Hxt7 was used as a negative control (Fig. 1d). The ability of the cells to grow was controlled on ethanol containing media (Fig. 1e) because AFY10 is unable to grow on maltose due to the deletion of all hexokinase genes^15^. Hxt11 and Hxt15 conferred similar growth rates on xylitol, but only after a seven days long lag phase. Interestingly, Hxt11 supports strong growth on glucose, in contrast to hexitol transporters (Supplementary Fig. S4), which suggests that xylitol transport is probably not its primary function.

### Kinetic properties and transport mechanism of polyol transporters

**Figure 2:**
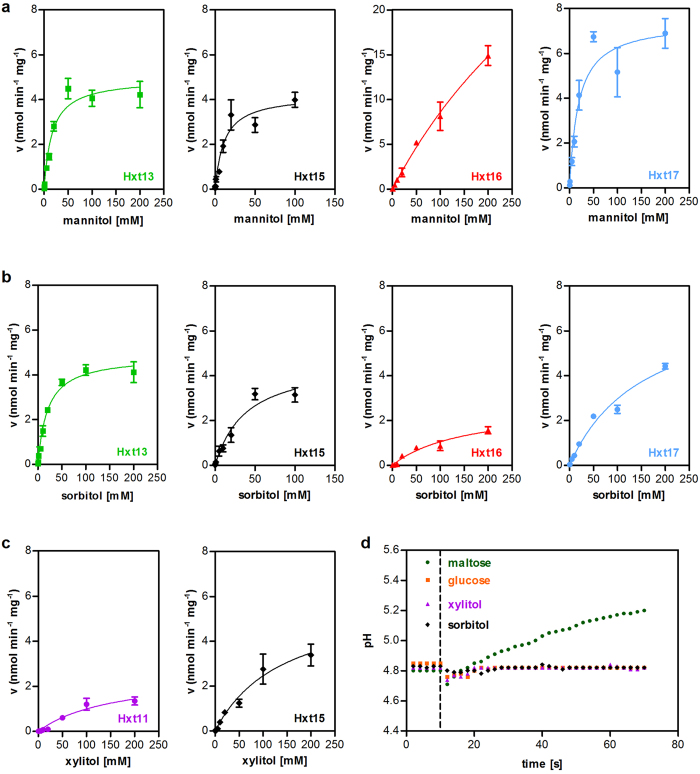
Uptake kinetics and transport mechanism. Individual transporters were co-expressed with the mannitol dehydrogenase YNR073C (**a**), sorbitol dehydrogenase *SOR1* (**b**) or xylitol dehydrogenase *XYL2* (**c**) in EBY.VW4000 (**a**,**b**) or AFY10 (**c**). The uptake velocity v (expressed as nmol polyol transported per minute per mg cell dry weight) is plotted against substrate concentration. Mean values and standard deviation were calculated from biological triplicates. The lines represent a least-square fit to the Michaelis-Menten equation. (**d**) Hxt1, Hxt11 or Hxt15 were individually overexpressed in EBY.VW4000. The change of the pH value in an unbuffered suspension of cells after addition of glucose (Hxt1), xylitol (Hxt11) or sorbitol (Hxt15) was measured. Maltose symport in the EBY.VW4000 background was used as a positive control. Dashed line indicates the time point of carbohydrate addition.

For determination of the kinetic properties of the identified polyol transporters, we performed uptake assays with radiolabeled substrates in strains expressing individual transporters and the appropriate polyol dehydrogenase. Hxt13, Hxt15 and Hxt17 exhibit similar affinities (reflected by the Michaelis constant, K_M_) and transport capacities (maximum velocity, v_max_) for mannitol. Hxt16 has a drastically lower affinity for mannitol compared to the other transporters, which is likely limiting its ability to confer growth on mannitol (Fig. 1a, Table 1). Hxt13 and Hxt15 have a moderate affinity for sorbitol, while the K_M_ value of Hxt16 and Hxt17 is approximately five times higher (Fig. 2b, Table 1). Faster growth rates of cells containing Hxt17 compared to those expressing Hxt16 can be explained by the higher v_max_ value of Hxt17. Overall, the kinetic parameters of the four polyol transporters correlate with growth rates conferred by them. Hxt15 seems to be the most versatile polyol transporter, while Hxt16 has a limited ability to confer growth on polyols due to its poor kinetic parameters.

The rather high K_M_ values of Hxt13, Hxt15, Hxt16 and Hxt17 for polyols suggest that they act as facilitators, like other members of the Hxt family in *S. cerevisiae*. To test this assumption experimentally, we measured the pH change of cell suspensions in unbuffered water upon addition of the carbohydrates. In the case of proton symport, the pH value is expected to rise, while a facilitative mechanism does not affect the proton concentration of the medium^19^,^20^. As a positive control, we used maltose, since EBY.VW4000 contains functional maltose transporters, which are known to act as symporters. After adding maltose, we observed a significant alkalinization of the suspension (Fig. 2d). In contrast, addition of glucose to the strain expressing *HXT1*, a known facilitator, did not cause a change of the extracellular pH. No pH changes were observed upon addition of xylitol and sorbitol to cells expressing Hxt11 and Hxt15, respectively. This supports the conclusion that they transport polyols by a facilitative mechanism.

### Ligand docking to Hxt structural models

To explore potential differences at the active site that can give insight into the observed substrate specificity among Hxt transporters, we generated structural models for Hxt transporters on the basis of crystal structures of Hxt homologues and examined the docking of mannitol, sorbitol and xylitol in these models.

**Figure 3:**
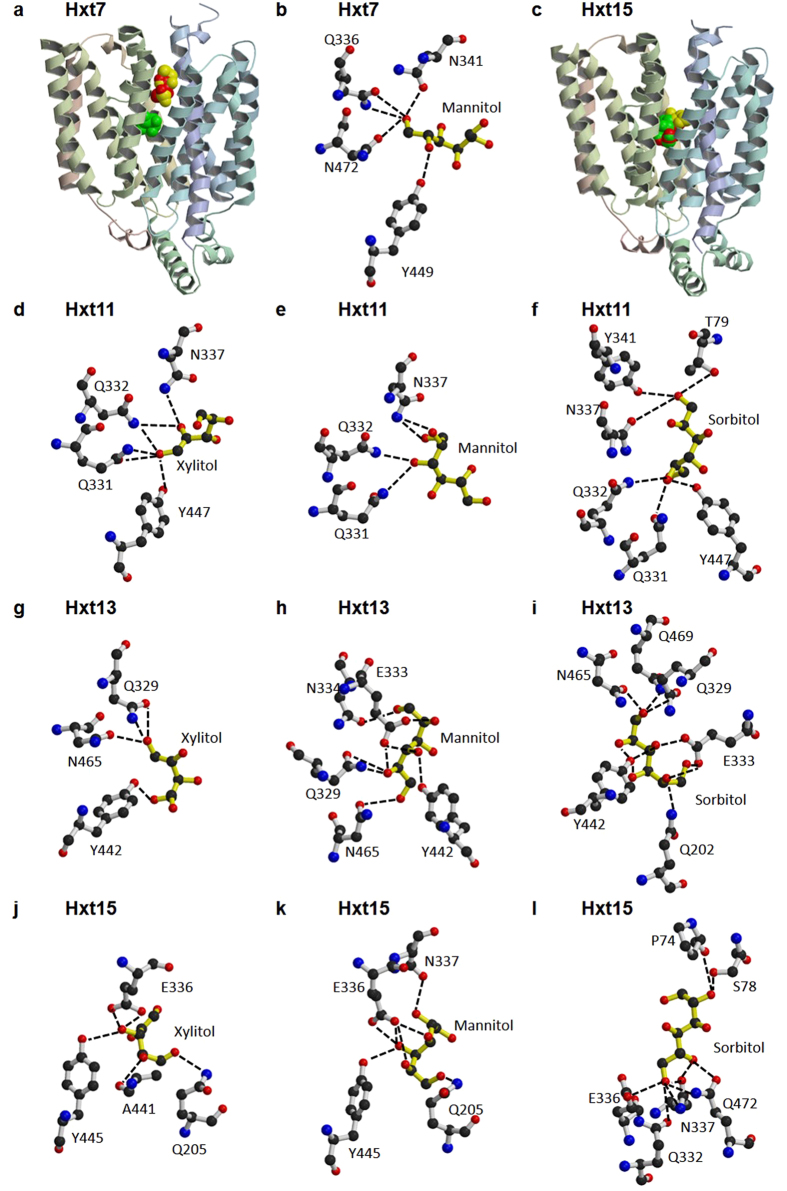
Ligand docking to Hxt7, Hxt11, Hxt13 and Hxt15 homology models. The dotted lines indicate hydrogen bonds. Overview of ligand docking to Hxt7 (**a**) or Hxt15 (**c**) structural models (cpk in yellow, red and green are sorbitol, xylitol and mannitol, respectively). (**b**) Predicted interactions of docked mannitol with active site residues of Hxt7. Predicted interactions of docked xylitol (**d**), mannitol (**e**) or sorbitol (**f**) with active site residues of Hxt11. Predicted interactions of docked xylitol (**g**), mannitol (**h**) or sorbitol (**i**) with active site residues of Hxt13. The same interactions are predicted for these ligands in Hxt17, as the active site residues are identical in Hxt13 and Hxt17. Predicted interactions of docked xylitol (**j**), mannitol (**k**) or sorbitol (**l**) with active site residues of Hxt15. The same interactions are predicted for these ligands in Hxt16, as the active site residues are identical in Hxt15 and Hxt16. Figure was generated with Molscript^38^ and Raster3D^39^.

**Figure 4:**
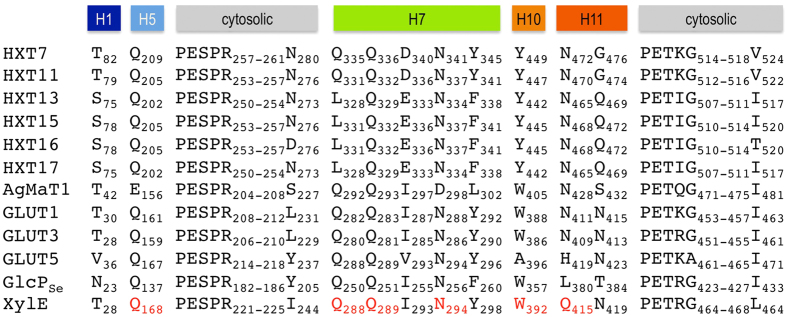
Sequence alignment for residues related to ligand binding. Included in the alignment are the sequences of Hxt homologues with known crystal structures: human glucose transporters (GLUT1, GLUT3 and GLUT5), *Escherichia coli* xylose/H+ symporter (XylE) and *Staphylococcus epidermidis* glucose/H+ symporter (GlcP_Se_). Plant polyol transporters are represented by *Apium graveolens* mannitol transporter AgMat1. In red are indicated the active site residues identified in the crystal structure of XylE (PDB ID 4GC0). Above the sequences are shown the corresponding transmembrane helices, colored as in Fig. 3a,c. The only difference between Hxt15 and Hxt16 sequences is in the positions of D276 and T520 of Hxt16, and come after strictly conserved cytosolic segments. Hxt13 and Hxt17 sequences are identical except for the first 15 amino acids in the N-terminus.

Sequence identity among Hxt proteins (Hxt7, Hxt11, Hxt13, Hxt15, Hxt16, Hxt17) and their closest homologues with determined crystal structures (XylE^21^, GlcP_Se_^22^, GLUT1^23^, GLUT3^24^ and GLUT5^25^) is shown in Supplementary Table S5. Among the crystal structures, while GlcP_Se_ has the highest sequence similarity to Hxt proteins, only XylE has a carbohydrate liganded structure. Therefore, XylE complexed with 6-bromo-6-deoxy-glucose (PDB ID 4GC0) was used as the basis for homology modeling of Hxt proteins. For the cytosolic regions between helices 3 and 4 that are highly variable between XylE and Hxt proteins, GlcP_Se_ structure (PDB ID 4LDS) was used instead. For all the structural models, the chosen docking region was the largest identified by Molecular Operating Environment (MOE, Chemical Computing Group), and coincided with the central cavity that houses the active site of other related sugar porters^21^,^22^,^23^,^24^,^25^. The docking experiments employed three ligands for each structural model: sorbitol, xylitol and mannitol. The results are illustrated in Fig. 3. Hxt13 and Hxt15 ligand interactions are representative for those in Hxt17 and Hxt16, respectively, as they have identical active sites and share high sequence identity (97% between Hxt13 and Hxt17, 99% between Hxt15 and Hxt16; Fig. 4 and Supplementary Table S5). First, there was a significant difference in the ligand docking between Hxt7 (the control transporter that does not transport polyols) and Hxt11, Hxt13 and Hxt15. In Hxt7, the three ligands were distributed throughout the central cavity, with sorbitol and xylitol putatively binding above the active site, and mannitol, though in the active site, interacting with protein residues just on one side of the molecule, with two of its hydroxyl groups (Fig. 3 a,b). On the other hand, in Hxt11, Hxt13 and Hxt15 all three ligands docked at the putative active site (Fig. 3c). Additionally, compared to Hxt7 (Fig. 3b), the interactions of docked mannitol with Hxt11 (Fig. 3e) are more complex and involve three hydroxyl groups of the ligand; in Hxt13 and Hxt15 four to five hydroxyl groups of mannitol form hydrogen bond interactions with protein residues (Fig. 3h,k). Docked sorbitol interacts with protein residues through two hydroxyl groups in Hxt11 (Fig. 3f) but three in Hxt15 (Fig. 3l) or six in Hxt13 (Fig. 3i), with an accompanying increase in the complexity of hydrogen bonding with protein residues. Finally, xylitol has the highest number of interactions in Hxt15 (3 hydroxyl groups involved in hydrogen bonds with protein residues; Fig. 3j). While in both Hxt11 and Hxt13 two hydroxyl groups of xylitol interact with protein residues, in Hxt11 there are more protein residues that anchor these groups, compared to Hxt13 (Fig. 3d,g). Figure 4 shows the sequence conservation for the active site residues of Hxt proteins predicted to interact with docked ligands (Fig. 3), compared to other homologous sugar porters with determined crystal structures. Expectedly, most of these residues have been implicated in hexose or pentose binding in Hxt homologues, such as GLUT1, GLUT3, GLUT5, XylE or GlcP_Se_^21^,^22^,^23^,^24^,^25^; for example Q202, N334, Q329, and N465 of Hxt13 (Fig. 4). There are also a couple of significant differences between the active sites of pentose/hexose transporters and those of Hxt polyol transporters. First, E333 of Hxt13 is conserved in Hxt15-17 or is an Aspartate in Hxt11, but is an Isoleucine in GLUT transporters, XylE or GlcP_Se_. This position is involved in hydrogen bond interactions with a hydroxyl group of mannitol or sorbitol in Hxt13 (Fig. 3h,i) or with mannitol, sorbitol or xylitol in Hxt15 (Fig. 3j–l). Second, Y442 of Hxt13 is strictly conserved in the other Hxt proteins but is a Tryptophan in GLUT1-4, XylE or GlcP_Se_. Interestingly, this position seems to be important in substrate specificity in human GLUTs^26^.

### Site-directed mutagenesis of Hxt15

**Figure 5:**
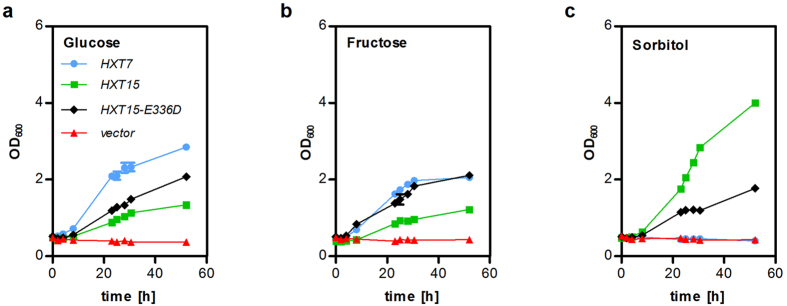
Growth assays with mutated Hxt15. SC-media supplemented with 2% (w/v) of the indicated carbon source were inoculated in shake flasks with EBY.VW4000 cells expressing the indicated transporter variants and the sorbitol dehydrogenase *SOR1*. Growth was monitored by measuring OD_600_ of the cultures. The results of duplicate measurements are shown.

Molecular modeling and docking pinpointed the conserved Glutamate corresponding to E336 of Hxt15 as a residue crucial for coordination of hexitol molecules. Therefore, we mutated E336 of Hxt15 to Aspartate, which is found at the corresponding position in *bona fide* hexose transporters, represented by Hxt7 in Fig. 4. In contrast to Hxt13, Hxt16 and Hxt17, Hxt15 shows a residual glucose transport activity (Supplementary Fig. S4; Fig. 5), besides being highly efficient as a sorbitol and mannitol transporter (Fig. 1). The specificity of the mutated protein Hxt15-E336D is reversed; its ability to confer growth on glucose (Fig. 5a) and fructose (Fig. 5b) is increased, while the transport of sorbitol becomes less efficient compared to wildtype Hxt15 (Fig. 5c). This underscores a pivotal role of the conserved Glutamate for discrimination between hexoses and hexitols in polyol transporters. The reverse mutation (D336E), however, did not convert Hxt11 to a hexitol transporter (data not shown), which suggests that more than one particular position is responsible for polyol transport.

### Specificity of polyol dehydrogenases

Hxt13, Hxt15, Hxt16 and Hxt17 showed comparable kinetics for both mannitol and sorbitol transport. To clarify whether (putative) mannitol and sorbitol dehydrogenases encoded in their genomic proximity show a similar substrate promiscuity, we determined their kinetic parameters for these polyols. *DSF1*, YNR073C, *SOR1* and *SOR2* were overexpressed from multicopy plasmids in CEN.PK2-1C grown on glucose, a carbon source that represses the endogenous genes of polyol utilization via the Tup1-Cyc8 corepressor complex^12^. Under these conditions, controls transformed with empty vectors instead of polyol dehydrogenase constructs showed neither mannitol nor sorbitol dehydrogenase activities in the crude protein extract. Thus, determination of overexpressed polyol dehydrogenase kinetics without further purification was feasible. The data are shown in Supplementary Fig. S6 and calculated K_M_ and v_max_ values are summarized in Table 2. Mannitol dehydrogenases YNR073C and Dsf1 have affinities for mannitol in the low mM range. Although they also show significant sorbitol dehydrogenase activities (Supplementary Fig. S6e,f), their K_M_ value for this polyol is by two orders of magnitude higher, which shows they are indeed specific for mannitol. Sorbitol dehydrogenases Sor1 and Sor2 have a K_M_ for sorbitol of approximately 15 mM and show only a very weak mannitol dehydrogenase activity (Supplementary Fig. S6d,e). Thus, the specificity of polyol dehydrogenases is significantly higher than that of polyol transporters. Based on the data presented here, we propose renaming *DSF1* and YNR073C genes to *MAN1* and *MAN2* (mannitol dehydrogenase 1 and 2, respectively).

## Discussion

The function of hexose transporter family members Hxt8-17 in *Saccharomyces cerevisiae* has been obscure for a long time. Here, we presented evidence that Hxt13, Hxt15, Hxt16 and Hxt17 transport mannitol and sorbitol with moderate affinities by a facilitative mechanism. The transport of polyols is likely to be their true function, since *HXT15* and *HXT16* genes are co-localized with sorbitol dehydrogenase genes *SOR2* and *SOR1*, while *HXT13* and *HXT17* are co-localized with *DSF1* and YNR073C, which encode mannitol dehydrogenases. Clustering of functionally related genes is often observed in *S. cerevisiae* (e.g. *MAL* and *GAL* genes for maltose and galactose utilization, respectively) and may reflect an evolutionary mechanism driven by the need for co-inheritance and co-regulation of genes involved in the utilization of non-preferred carbon sources. Of all transporters tested, only Hxt11 and Hxt15 were capable of moderate xylitol transport. It is unlikely that Hxt11 is primarily a xylitol transporter because it can transport a broad range of substrates including hexoses glucose, fructose, mannose and galactose^10^, as well as the pentose xylose (M. Oreb, unpublished result). In addition, Hxt11 (and its paralogue Hxt9) have been implicated in drug resistance processes mediated by transcription factors Pdr1 and Pdr3^13^. On the other hand, Hxt15 is a *bona fide* candidate for a xylitol permease, having a higher transport capacity for the pentitol compared to Hxt11 (Fig. 2c, Table 1, Supplementary Fig. S3). Moreover, sorbitol dehydrogenases are known to accept xylitol as a substrate^6^.

Ligand docking to the homology models of Hxt transporters predicted that the substrate binding site is located in the internal part of the central cavity, similarly as in Hxt homologues such as human GLUTs or bacterial GlcP_Se_ and XylE. Generally the complexity of protein-ligand interactions from docking experiments mirrors the substrate affinity and specificity observed in the transport assay (Table 1). For example, in the case of Hxt15 K_M_ for mannitol is ~13-fold lower than that of xylitol (Table 1). Correspondingly, mannitol has 5 (out of 6) hydroxyl groups engaged in 7 possible hydrogen bonds with active site residues (Fig. 3k) while xylitol has 3 (out of 5) hydroxyl groups making 5 possible hydrogen bonds with almost the same residues (Fig. 3j). The same trend is observed when a particular ligand is examined across Hxt proteins. For instance, xylitol is transported with comparable affinity (K_M_, Table 1) in Hxt11 and Hxt15, but is not a substrate for Hxt13. Accordingly, the network of hydrogen bond interactions (Fig. 3 d,g,j) increases as the K_M_ for substrate decreases (Table 1). The correspondence between the observed interactions in the ligand docking experiments and the kinetic parameters of transport holds for Hxt7, Hxt11, Hxt13 and Hxt15, but breaks down when comparing Hxt13 and Hxt17 or Hxt15 and Hxt16. Hxt13 and Hxt17 have similar K_M_ values for mannitol, but have an 8-fold difference in the K_M_ for sorbitol (Table 1). When comparing Hxt15 and Hxt16, K_M_ for both mannitol and sorbitol are significantly higher (~50- and 4-fold, respectively) for Hxt16. Nonetheless, the active sites of either Hxt13 and Hxt17 or Hxt15 and Hxt16 are identical, with each pair of transporters sharing more than 97% sequence identity (Supplementary Table S5). Thus, Hxt13 and Hxt17 differ in sequence only in the N-terminal 15 amino acids, while Hxt15 and Hxt16 differ by only two aminoacids. Interestingly, these two residues (D276 and T520 of Hxt16) are located after highly conserved cytosolic segments (Fig. 4). This raises the possibility of long-range influence on the transport activity/specificity. The cytosolic modules are implicated in gating the sugar translocation pathway and modulating conformational changes between the outward and inward open conformations of the related XylE transporter^21^,^27^. This suggests that transporter dynamics, rather than substrate binding, is responsible for distinct properties of Hxt15 and Hxt16. A dramatic example of long-range effects is illustrated by the 200-fold decrease of the K_d_ for lactose when *E. coli* lactose permease (LacY) has a nanobody bound to its periplasmic region^28^. LacY and Hxt transporters belong to the major facilitator superfamily (MFS), whose members are believed to share an alternating mechanism of substrate transport^29^. Therefore, identifying regions responsible for long-range modulation of transport activity can be relevant to many other MFS proteins.

Analysis of the modeled ligand binding to the active sites of Hxt polyol transporters pinpoints two Hxt-specific residues: those corresponding to E336 and Y445 in Hxt15. These residues are conserved in Hxt polyol transporters and are involved in hydrogen bond interactions with the hydroxyl groups of mannitol, sorbitol or xylitol (Fig. 3). Mutating E336 of Hxt15 to Aspartate reverses the specificity from hexitols to hexoses (Fig. 5), which confirms the pivotal role of this position in substrate recognition. However, the mutation D336E does not convert Hxt11 to a hexitol transporter (data not shown), which shows that hexitol transport is dependent on more than one particular residue. The identity of these residues has to be established by future studies. The residue corresponding to Y445 in Hxt15 is capable to form hydrogen bond interactions with xylitol in Hxt11 or Hxt15/Hxt16, with mannitol in Hxt13/Hxt17 or Hxt15/Hxt16, and also with sorbitol in Hxt13/Hxt17 or Hxt15/Hxt16 (Fig. 3). Tyr in this position does not seem to discriminate among the polyol ligands and is also present in low/high affinity hexose transporters like Hxt1-7, all of which transport glucose, fructose and mannose^7^. Nevertheless, this position is important for substrate specificity in human GLUTs^26^. Thus when A396 of GLUT5, a fructose-only transporter, was mutated to Tryptophan, as in human GLUTs that transport glucose, GLUT5 A396W gained the capability to transport glucose, without compromising the transport activity. We speculate that Tyr in this position contributes to substrate promiscuity in Hxt transporters, which may be desirable in yeast.

Hxt13, Hxt15, Hxt16 and Hxt17 are structurally and functionally distinct from other known polyol transporters. In bacteria, phosphotransferase systems for mannitol transport have been described^30^. Plant mannitol and sorbitol transporters, represented by AgMaT1^31^ and PcSOT1^4^, as well as polyol transporters from the halotolerant yeast *Debaryomyces hansenii*^20^, act as proton symporters, which is mirrored by their lower K_M_ values (in the sub-mM range). They show little conservation of residues involved in the canonical substrate binding pocket compared to Hxts (see AgMaT1 in Fig. 4). Hxt polyol transporters are phylogenetically more closely related to hexose/pentose transporters (including bacterial and mammalian homologs; Supplementary Fig. S7) than to polyol transporters from other eukaryotic species. Like other yeast hexose transporters, they act as facilitators (Fig. 2d). Therefore, Hxt13, Hxt15, Hxt16 and Hxt17 can be assigned as a novel type of polyol transporters. Since they clearly act as hexitol and not as hexose transporters, we propose renaming them to Hlt1-4 (hexitol transporter 1–4).

It is intriguing that yeast cells grow on hexitols only after long adaptation^5^,^6^,^11^, although their genomes encode active mannitol and sorbitol dehydrogenases^5^,^6^ (see also Supplementary Fig. S6) as well as *bona fide* polyol transporters (this study). One possible reason for this cryptic behavior could be their localization in subtelomeric regions, which are normally silenced. At the same time, subtelomeres are extraordinarily unstable and act as hot spots for evolution of new functions within families of duplicated genes^40^. Based on phylogenetic analysis (Supplementary Fig. S7) and mutagenesis experiments (Fig. 5), it is feasible to assume that the hexitol transporter function arose from mutations in duplicated hexose transporters. Only after prolonged selective pressure^5^,^6^,^11^, which is often accompanied by mutations within repressor genes^12^, the subtelomeric genes for hexitol dehydrogenases and hexitol transporters become derepressed, which enables the cells to utilize these non-preferred carbon sources.

## Materials and Methods

### Yeast strains and growth conditions

The strains used in this study are listed in Supplementary Information table S1. Construction of strains EBY.VW4000^10^ and AFY10^15^ was described previously. Plasmid-free strains were grown in standard YEP-media supplemented with maltose (EBY.VW4000) or ethanol (AFY10). Frozen competent cells were prepared and transformed according to the established protocol^32^. Strains transformed with plasmids were cultivated on synthetic complete (SC) medium^33^ supplemented with the appropriate carbon source (2% w/v), whereby tryptophan and histidine were added as required by the combination of auxotrophic markers. All growth assays were performed at 30 °C with shaking at 180 rpm.

### Plasmid construction

The protein coding sequences of all genes used in this study are as annotated for the S288C reference strain in the Saccharomyces Genome Database (SGD; http://www.yeastgenome.org). Due to the high sequence similarity of some isoform pairs, a two-step PCR protocol was necessary to enrich the desired paralogue selectively. The first primer pair was targeted to promoter and terminator regions that were divergent between the isoforms. In a second PCR, the coding sequence was amplified from the first PCR product. This procedure was used to discriminate between *HXT9*/*HXT11* and *DSF1*/YNR073C. The sequences of paralogous pairs *HXT15*/*HXT16* and *SOR1*/*SOR2* are highly similar even in the promoter and terminator regions, so that we could not specifically enrich them by nested PCR as described above. Therefore, the correct *SOR1* and *SOR2* clones were identified randomly by sequencing after plasmid construction. The *HXT16* sequence was generated by site directed mutagenesis of *HXT15* (the triplets encoding D276 and T520 were introduced according to the *HXT16* SGD-entry) because no clone with a sequence corresponding to the *HXT16* entry could be found. The primers used in this study are listed in Supplementary Information table S2. All ORFs were inserted between the truncated *HXT7* promoter and *CYC1* terminator sequences of p42XH7 series vectors by the standard gap-repair procedure. Transporter genes were cloned into p426H7 (2 μ, *URA3*) and polyol dehydrogenases into p425H7 (2 μ, *LEU2*). The construction of *HXT1*, *HXT4*, *HXT10* and *HXT14* plasmids was reported previously^34^.

### Uptake assays with radiolabeled polyols

D−[1−^14^C] mannitol (55 mCi/mmol), D−[^14^C(U)] sorbitol (300 mCi/mmol) and D−[1−^3^H] xylitol were obtained from American Radiolabeled Chemicals, Inc. (St. Louis, MO, USA).

EBY.VW4000 and AFY10 cells transformed with plasmids as indicated in the results section were pre-grown in selective SCM and SCE medium, respectively. The polyol concentration in the uptake mixture was varied between 0.5 and 200 mM, whereby the proportion of the radiolabeled polyol was 0.14 μCi to 0.61 μCi per sample. The samples were incubated for 10 s at 30 °C before the uptake was stopped in ice-cold buffer containing 500 mM non-labeled polyol. Background binding was controlled by separately pipetting cells transformed with the empty vectors and radiolabeled polyol solutions into the quenching buffer. Other technical details were as previously described^15^. The kinetic parameters (K_M_, v_max_) were calculated by a least-square fit to the Michaelis-Menten equation using GraphPad Prism software, version 5.01.

### Protein extraction and enzyme assays

For enzyme assays in total protein extracts, 50 ml of an exponentially growing culture were harvested by centrifugation and the cell pellets were washed in the appropriate assay buffer. The cells were mechanically disrupted by shaking (10 min at 4 °C) with glass beads (0.45 mm diameter) using a Vibrax cell disruptor (Janke & Kunkel, Staufen, Germany) and the cell debris was subsequently removed by centrifugation (15.000xg, 5 min, 4 °C). Protein concentration of clear crude extracts was determined by the Bradford method, using bovine serum albumin as a standard. Enzyme assays were performed in 200 μl containing 60 mM HEPES-NaOH (pH 7.4), 1.66 mM NAD^ + ^, and variable concentrations of mannitol or sorbitol. The reaction was started by adding 10 μl of the cell lysate. The reduction of NAD^+^ was monitored in a Ultrospec 2100 pro spectrometer.

### Generation of Hxt structural models and ligand docking

Blast search against Protein Data Bank (PDB) for Hxt transporters showed that GlcP_Se_ (PDB ID 4LDS)^22^ has the highest sequence identity to Hxt transporters (Supplementary table S5), closely followed by GLUT3^24^, GLUT1^23^, XylE^21^ and GLUT5^25^. Among these, crystal structures of GLUT3 and XylE contained ligands, however, GLUT3 also included lipids at the active site from the crystallization. Therefore, structural models for Hxt7, Hxt11, Hxt13, Hxt15, Hxt16 and Hxt17 were generated with Coot^35^, on the basis of XylE outward facing structure (PDB ID 4GC0)^21^, supplemented where needed through comparison with the crystal structure of GlcP_Se_^22^. Amino acid replacement in the models was guided by sequence alignment between XylE or GlcP_Se_ and the modeled Hxt protein, as determined by ClustalW^36^. Resulting structural models underwent energy minimization with Phenix^37^ and then with Molecular Operation Environment software (MOE, Chemical Computing Group).

The docking of mannitol, sorbitol and xylitol to the Hxt models was performed with MOE. All Hxt models were prepared for docking in the same way: using Protonate 3D (pH 7.5) and Energy Minimize functions. The putative ligand binding sites were determined with SiteFinder; the first and largest site identified with this program corresponded to the general area for substrate binding in other sugar porters and Hxt homologues^21^,^22^,^24^, and was selected for ligand docking. Possible conformations for the ligands (mannitol, sorbitol and xylitol) were generated with Conformational Generation function. Ligand docking was performed with Dock function, with default parameters in Triangle Matcher, Alpha HB rescoring, and retaining 30 poses. Potential ligand docking positions were further selected if they had sufficient space for ligand binding, polar or charged residues, and low energy of interaction as indicated by the lowest-energy scoring algorithm.

### Symport assay

The symport assay was conducted by measuring the alkalinization of unbuffered cell suspensions upon substrate addition as previously described^19^. 200 OD_600_-Units of EBY-VW4000 cells transformed with the appropriate plasmid were harvested from a culture exponentially growing on SCM-Ura medium and resuspended in 1 ml of water adjusted to pH 4.8 with HCl. The cells were pipetted into 23 ml of water, pH 4.8 and the baseline was recorded before the uptake was started by adding 1 ml of a 50% (w/v) solution of appropriate carbohydrate, adjusted to pH 4.8. Alkalinization was measured in a 50 ml beaker at 22 °C.

## Additional Information

**How to cite this article**: Jordan, P. *et al.* Hxt13, Hxt15, Hxt16 and Hxt17 from *Saccharomyces cerevisiae* represent a novel type of polyol transporters. *Sci. Rep.* **6**, 23502; doi: 10.1038/srep23502 (2016).

## Supplementary Material

Supplementary Information

## Figures and Tables

**Table 1:** Kinetic parameters of polyol transporters.

	Mannitol			Sorbitol			Xylitol		
	μ_max_^1^	v_max_^2^	K_M_^2^	μ_max_^1^	v_max_^2^	K_M_^2^	μ_max_^1^	v_max_^2^	K_M_^2^
Hxt11	n.d.	n.d.	n.d.	n.d.	n.d.	n.d.	0.09 ± 0.01	2.6 ± 0.5	159.1 ± 57.8
Hxt13	0.21 ± 0.01	4.9 ± 0.2	16.7 ± 2.9	0.22 ± 0.01	4.8 ± 0.2	20.4 ± 2.4	n.d.	n.d.	n.d.
Hxt15	0.20 ± 0.01	4.2 ± 0.3	11.4 ± 2.7	0.22 ± 0.01	4.7 ± 0.5	38.9 ± 8.6	0.09 ± 0.01	5.9 ± 0.9	143.3 ± 40.9
Hxt16	0.12 ± 0.03	53.8 ± 13.1	527.6 ± 166.1	0.10 ± 0.01	2.7 ± 0.4	152.8 ± 47.1	n.d.	n.d.	n.d.
Hxt17	0.22 ± 0.02	7.4 ± 0.5	18.6 ± 4.3	0.21 ± 0.01	7.6 ± 0.8	155.7 ± 29.5	n.d.	n.d.	n.d.

n.d. – not determined.

^1^calculated from data presented in Fig. 1; μ_max_ values are given in h^−1.^

^2^calculated from data presented in Fig. 2; v_max_ values are given in nmol substrate transported per minute per mg dry cell weight; K_M_ values are in mM.

**Table 2:** Kinetic parameters of polyol dehydrogenases.

	Mannitol		Sorbitol	
	v_max_^1^	K_M_^1^	v_max_^1^	K_M_^1^
YNR073C	0.40 ± 0.02	3.6 ± 0.5	2.1 ± 0.5	634.9 ± 207.1
Dsf1	0.90 ± 0.02	2.9 ± 0.3	1.5 ± 0.7	869.6 ± 601.8
Sor1	0.10 ± 0.02	89.0 ± 37.5	0.40 ± 0.01	11.8 ± 1.4
Sor2	0.05 ± 0.01	98.4 ± 25.7	0.5 ± 0.02	15.1 ± 2.8

v_max_ values are given as μmol substrate converted per minute per mg of total protein; K_M_ values are in mM.

^1^calculated from data presented in Supplementary Fig. S6.
