# Supplementary material for: Hxt13, Hxt15, Hxt16 and Hxt17 from Saccharomyces cerevisiae represent a novel type of polyol transporters
Source: Sci Rep. 2016 Mar 21;6:23502. doi: 10.1038/srep23502 (PMC4800717; doi:10.1038/srep23502)
Supplement: Supplementary Information [file srep23502-s1.pdf]

Supplementary Information

Hxt13, Hxt15, Hxt16 and Hxt17 from *Saccharomyces cerevisiae* represent a novel type of polyol transporters

Paulina Jordan, Jun-Yong Choe, Eckhard Boles & Mislav Oreb

Supplementary Table S1: Strains used in this study

Strain name	Relevant genotype	References
EBY.VW4000	*MATa leu2-3,112 ura3-52 trp1-289 his3-1 MAL2-8c SUC2 Δhxt1-17 Δgal2 Δstl1::loxP Δagt1::loxP Δmph2::loxP Δmph3::loxP*	[1]
AFY10	*EBY.VW4000 background; Δglk1::loxP Δhxk1::loxP Δhxk2::loxP Δylr446w::loxP pyk2Δ::pPGK1-opt.XKS1-tPGK1 pTPI1-TAL1-tTAL1 pTDH3-TKL1-tTKL1 pPFK1-RPE1-tRPE1 pFBA-RKI1-tRKI1 loxP*	[2]
CEN.PK2-1C	*MATa leu2-3,112 ura3-52 trp1-289 his3-1 MAL2-8c SUC2*	EUROSCARF

Supplementary Table S2: Primers used in this study

Target Gene	Primer Name	Sequence (5' - 3')	Application
HXT7	Hxt7f2	ATAAACACAAAAACAAAAAGTTTTTTTAATTTTAATC AAAAAATGTCACAAGACGCTGCTATTG	forward primer for *HXT7* cloning
	Hxt7r2	GGAGGGCGTGAATGTAAGCGTGACATAACTAATTACAT GACTCGAGTTATTTGGTGCTGAACATTCTC	reverse primer for *HXT7* cloning
HXT8	Hxt8_OV-p426H7prom_fw	AACACAAAAACAAAAAGTTTTTTTAATTTTAATCAAA AAATGACTGATCGTAAAACCAAC	forward primer for *HXT8* cloning
	Hxt8_OV-p426H7term_rev	GAATGTAAGCGTGACATAACTAATTACATGACTCGAGC TAAAACATTCTTTTGTAGAAGGG	reverse primer for *HXT8* cloning
HXT9, HXT11	Hxt9_ATG_fw	ATGTCCGGTGTTAATAATAC	forward primer for *HXT9* and *HXT11* amplification, binding in the promotor region of both genes (first PCR)
	Hxt9_Hxt9+1kb_rev	TGTCAACTCGTGTAGCTC	reverse primer for *HXT9* amplification, binding in the terminator region (first PCR)
	Hxt9_OV-p426H7prom_fw	AAAAACAAAAAGTTTTTTTAATTTTAATCAAAAAATG TCCGGTGTTAATAATACATCC	forward primer for *HXT9* cloning, (second PCR)

	Hxt11_Hxt11+1kb_rev	TGCACGTGACACTCACC	reverse primer for *HXT11* amplification, binding in the terminator region (first PCR)
	Hxt11_OV-p426H7prom_fw	AAAAACAAAAAGTTTTTTAATTTTAATCAAAAAATG TCAGGTGTTAATAATACATCC	forward primer for *HXT11* amplification (second PCR)
	Hxt11_OV-p426H7term_rev	GAATGTAAGCGTGACATAACTAATTACATGACTCGAGT CAGCTGGAAAAGAACCTC	reverse primer for *HXT9* and *HXT11* cloning (second PCR)
HXT13	Hxt13_OV-p426H7prom_fw	AACACAAAAACAAAAAGTTTTTTAATTTTAATCAAA AAATGTCTAGTGCGCAATCCTCTATTG	forward primer for *HXT13* cloning
	Hxt13_OV-p426H7term_rev	GAATGTAAGCGTGACATAACTAATTACATGACTCGAGT CAATCAGAATTCTTTGAGAACTTCAA	reverse primer for *HXT13* cloning
HXT15	Hxt15_OV-p426H7prom_fw	AACACAAAAACAAAAAGTTTTTTAATTTTAATCAAA AAATGGCAAGCGAACAGTCC	forward primer for *HXT15* and *HXT16* cloning
	Hxt15_OV-p426H7term_rev	GAATGTAAGCGTGACATAACTAATTACATGACTCGAGT CAATTAAAACTCTTTGGGAACTTC	reverse primer for *HXT15* and *HXT16* cloning
	Hxt15-E336D fw	TTGCAACTTACTGGTGACAACTACTTCTTCTTC	forward primer for introducing E336D into *HXT15* ORF
	Hxt15-E336D rev	GAAGAAGAAGTAGTTGTCACCAGTAAGTTGCAA	reverse primer for introducing E336D into *HXT15* ORF
HXT16	Hxt16_Mut1_rev	GTGAAACCTTGTCGATCTTGGCGATG	reverse primer for introducing N276D into *HXT15* ORF
	Hxt16_Mut1_fw	CATCGCCAAGATCGACAAGGTTTCAC	forward primer for introducing N276D into *HXT15* ORF
	Hxt16_Mut2_rev	CATATAGTAACTGGGTTTCCTCCAAAGATAG	reverse primer for introducing I520T into *HXT15* ORF
	Hxt16_Mut2_fw	CTATCTTTGGAGGAAACCCAGTTACTATATG	forward primer for introducing I520T into *HXT15* ORF
HXT17	Hxt17_OV-p426H7prom_fw	AACACAAAAACAAAAAGTTTTTTAATTTTAATCAAA AAATGCAATCATCCACTGAAAG	forward primer for *HXT17* cloning
	Hxt17_OV-p426H7term_rev	GAATGTAAGCGTGACATAACTAATTACATGACTCGAGT CAATCAGAACCCTTTGAGAAC	reverse primer for *HXT17* cloning
DSF1, YNR073C	YNR073C_YNR+1kb_fw	ATTCTGCTGTGGTGGTCG	forward primer for *YNR073C* amplification, binding in the promotor region (first PCR)
	YNR073C_YNR+1kb_rev	TTTGACTATCGATTGAAAATCATG	reverse primer for *YNR073C* amplification, binding in the terminator region (first PCR)
	YNR073C_OV_p425prom_fw	AACACAAAAACAAAAAGTTTTTTAATTTTAATCAAA AAATGACAAAATCAGACGAAAC	forward primer for *DSF1* and *YNR073C* cloning (second PCR)
	YNR073C_OV_p425term_rev	GAATGTAAGCGTGACATAACTAATTACATGACTCGAGT CACACTTGGTCTAAAATTTC	reverse primer for *DSF1* and *YNR073C* cloning (second PCR)
	DSF1-up_fw	ACGGACCTAAAAATAACTGTAG	forward primer for *DSF1* amplification, binding in the promotor region (first PCR)
	DSF1-down_rev	TTGACGATCGATTAAAGATC	reverse primer for *DSF1* amplification, binding in the terminator region (first PCR)
SOR1, SOR2	SOR1_OV-p425H7prom_fw	AACACAAAAACAAAAAGTTTTTTAATTTTAATCAAA AAATGTCTCAAAATAGTAACCCTGC	forward primer for *SOR1* and *SOR2* cloning
	SOR1_OV-p425term_rev	GAATGTAAGCGTGACATAACTAATTACATGACTCGAGT CATTCAGGACCAAAGATAATAG	reverse primer for *SOR1* and *SOR2* cloning

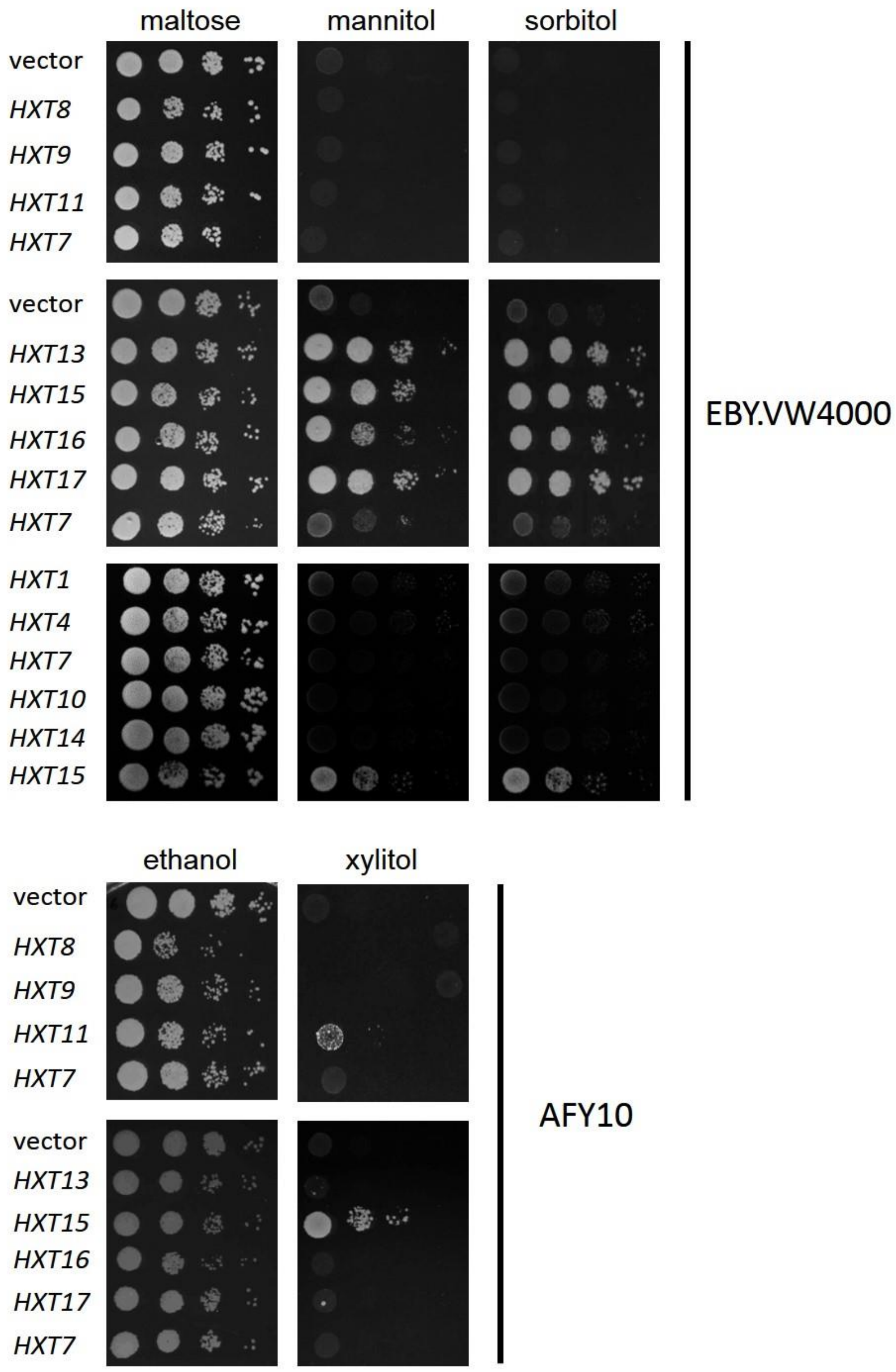

Supplementary Figure S3: Prescreening of individual transporters on polyols. Serial dilutions of transformants were dropped on SC media supplemented with 2% of the indicated carbon source. The plates were incubated for three days (EBY.VW4000) or seven days (AFY10) at 30°C.

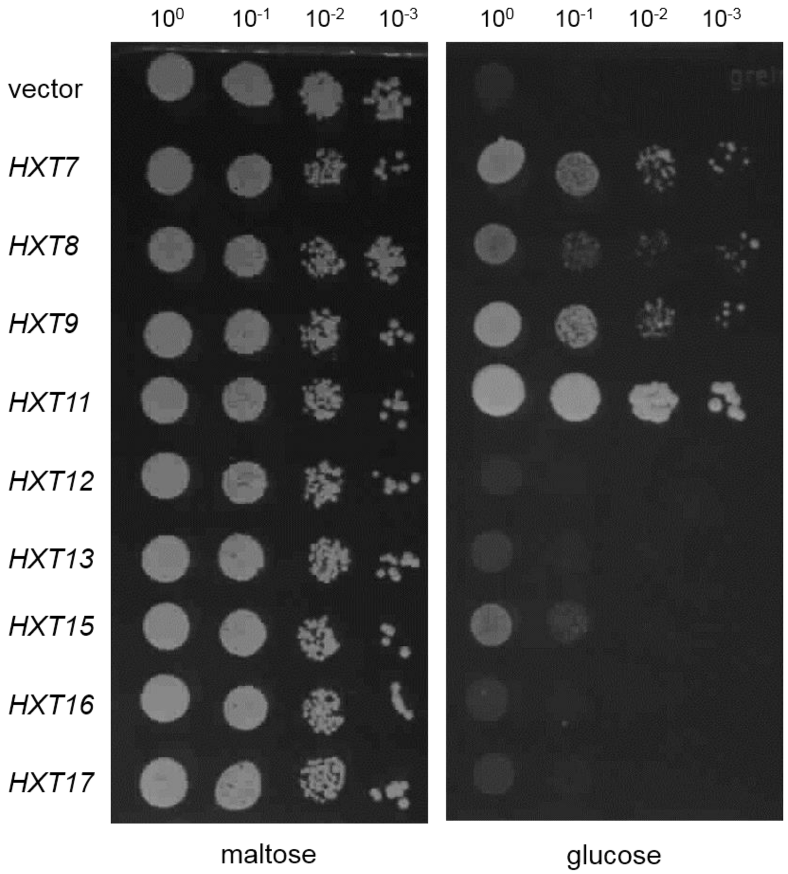

Supplementary Figure S4: Growth of EBY.VW4000 expressing individual transporters on glucose. Serial dilutions of transformants were dropped on SC media supplemented with 2% of the indicated sugar. Empty vector was used as a negative control and *HXT7* as a positive control for growth on glucose. The plates were incubated at 30°C for three days.

Supplementary Table S5: Sequence identities among Hxt7, Hxt11, Hxt13, Hxt15, Hxt16, Hxt17, AgMaT1, GLUT1, GLUT3, GLUT5, $GlcP_{Se}$, and XylE. The sequence identity was calculated with ClustalW[3] and SIAD (http://imed.med.ucm.es/Tools/sias.html).

**Hxt11**	72.49%										
**Hxt13**	59.22%	55.66%									
**Hxt15**	58.89%	54.69%	91.42%								
**Hxt16**	58.73%	54.53%	91.10%	99.67%							
**Hxt17**	58.73%	55.17%	97.57%	90.93%	90.61%						
**AgMaT1**	22.16%	22.49%	24.11%	22.81%	22.65%	24.11%					
**GLUT1**	25.88%	25.88%	25.56%	24.59%	24.43%	25.40%	34.14%				
**GLUT3**	26.86%	26.21%	26.05%	25.56%	25.40%	25.88%	32.52%	70.38%			
**GLUT5**	22.15%	20.75%	20.55%	20.35%	20.15%	20.55%	19.76%	41.26%	39.11%		
**$GlcP_{Se}$**	27.18%	27.02%	30.42%	29.44%	29.28%	30.42%	40.12%	41.10%	39.96%	27.31%	
**XylE**	23.30%	23.46%	22.16%	21.52%	21.52%	22%	32.03%	36.56%	36.08%	22.40%	41.58%
	**Hxt7**	**Hxt11**	**Hxt13**	**Hxt15**	**Hxt16**	**Hxt17**	**AgMaT1**	**GLUT1**	**GLUT3**	**GLUT5**	**$GlcP_{Se}$**

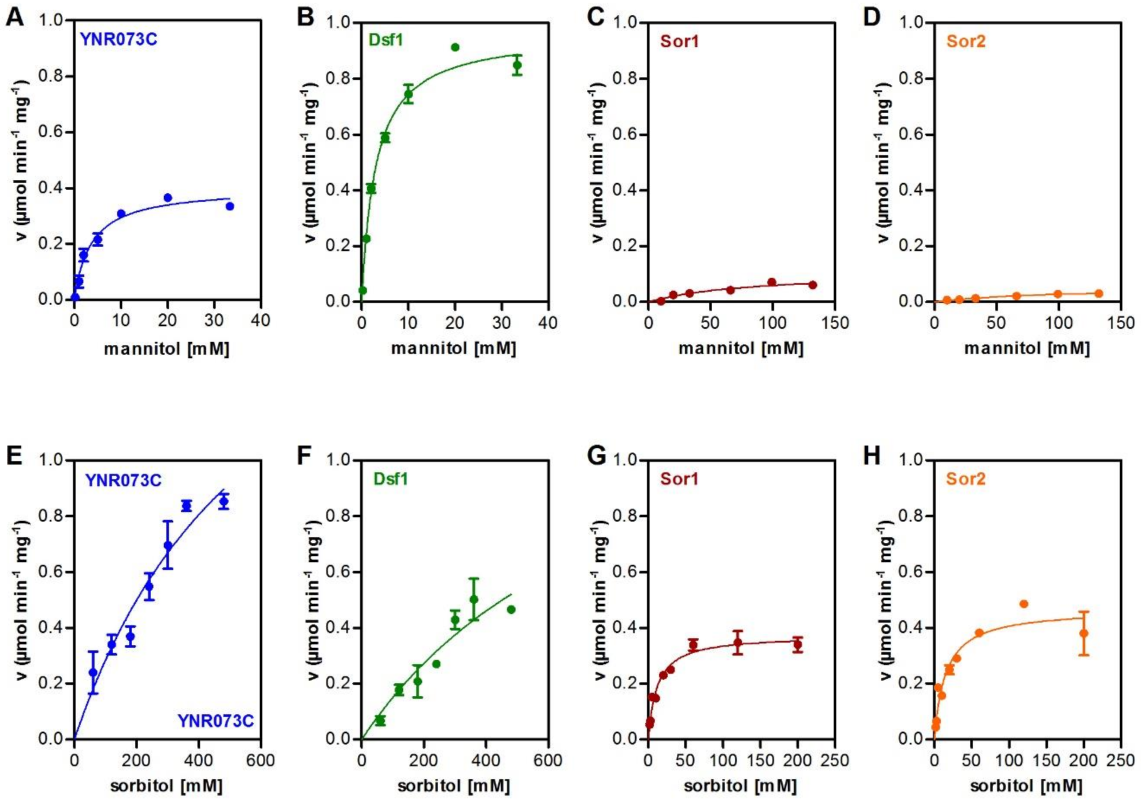

Supplementary Figure S6: Characterization of polyol dehydrogenases. Individual polyol dehydrogenases were overexpressed in CEN.PK2-1C grown on selective SC media containing glucose. The velocity (expressed as µmol substrate converted per minute per mg of total protein) of mannitol (A-D) and sorbitol (E-H) oxidation was measured in total protein extracts and plotted against the substrate concentration. Mean values and standard deviation were calculated from biological triplicates. Lines represent a least-square fit to the Michaelis-Menten equation.

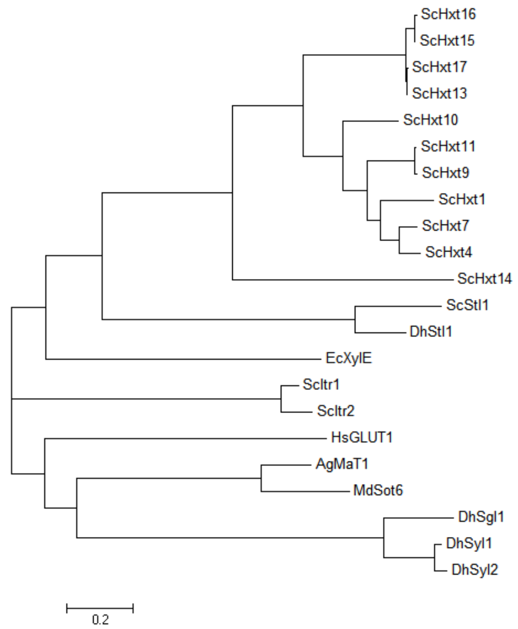

Supplementary Figure S7: **Phylogenetic relationships between selected polyol and sugar transporters.** Represented proteins are: *Apium graveolens* mannitol transporter AgMat1; *Debaryomyces hansenii* acyclic polyol transporters DhSyl1 and DhSyl2, galactitol transporter DhSgl1, glycerol transporter DhStl1; *E. coli* xylose transporter EcXylE; human glucose transporter HsGLUT1; *Malus domestica* sorbitol transporter MdSot1; *S. cerevisiae* hexose transporters family members ScHxt1, ScHxt4, ScHxt7, ScHxt9, ScHxt10, ScHxt11, ScHxt13, ScHxt14, ScHxt15, ScHxt16 and ScHxt17; inositol transporters ScItr1 and ScItr2; glycerol transporter ScStl1. The alignment was generated using ClustalW and the dendogram was calculated with the MEGA software[4], version 6 using the neighbour-joining method.

Supplementary References

1. Wieczorke, R. *et al.* Concurrent knock-out of at least 20 transporter genes is required to block uptake of hexoses in Saccharomyces cerevisiae. *FEBS Lett.* **464,** 123–128 (1999).
2. Farwick, A., Bruder, S., Schadeweg, V., Oreb, M. & Boles, E. Engineering of yeast hexose transporters to transport D-xylose without inhibition by D-glucose. *Proc. Natl. Acad. Sci. USA* **111,** 5159–5164 (2014).
3. Larkin, M. A. *et al.* Clustal W and Clustal X version 2.0. *Bioinformatics* **23,** 2947–2948 (2007).
4. Tamura, K., Stecher, G., Peterson, D., Filipski, A. & Kumar, S. MEGA6: Molecular Evolutionary Genetics Analysis version 6.0. *Mol. Biol. Evol.* **30,** 2725–2729 (2013).
